# Differential expression of slow and fast-repriming tetrodotoxin-sensitive sodium currents in dorsal root ganglion neurons

**DOI:** 10.3389/fnmol.2023.1336664

**Published:** 2024-01-11

**Authors:** Zhi-Yong Tan, Bin Wu, Xiaolin Su, You Zhou, Yong-Hua Ji

**Affiliations:** ^1^Department of Pathophysiology, Hebei University School of Basic Medicine, Baoding, China; ^2^Stark Neurosciences Research Institute, Indiana University School of Medicine, Indianapolis, IN, United States; ^3^Institute of Special Environment Medicine, Nantong University, Nantong, China; ^4^Department of Physiology, Hebei University School of Basic Medicine, Baoding, China

**Keywords:** Nav1.7, TTX-sensitive, slow-repriming, dorsal root ganglion, isolectin B4, pain

## Abstract

Sodium channel Nav1.7 triggers the generation of nociceptive action potentials and is important in sending pain signals under physiological and pathological conditions. However, studying endogenous Nav1.7 currents has been confounded by co-expression of multiple sodium channel isoforms in dorsal root ganglion (DRG) neurons. In the current study, slow-repriming (SR) and fast-repriming (FR) tetrodotoxin-sensitive (TTX-S) currents were dissected electrophysiologically in small DRG neurons of both rats and mice. Three subgroups of small DRG neurons were identified based on the expression pattern of SR and FR TTX-S currents. A majority of rat neurons only expressed SR TTX-S currents, while a majority of mouse neurons expressed additional FR TTX-S currents. ProTx-II inhibited SR TTX-S currents with variable efficacy among DRG neurons. The expression of both types of TTX-S currents was higher in Isolectin B4-negative (IB_4_^−^) compared to Isolectin B4-positive (IB_4_^+^) neurons. Paclitaxel selectively increased SR TTX-S currents in IB_4_^−^ neurons. In simulation experiments, the Nav1.7-expressing small DRG neuron displayed lower rheobase and higher frequency of action potentials upon threshold current injections compared to Nav1.6. The results suggested a successful dissection of endogenous Nav1.7 currents through electrophysiological manipulation that may provide a useful way to study the functional expression and pharmacology of endogenous Nav1.7 channels in DRG neurons.

## Introduction

Voltage-gated sodium channels (VGSCs) initiate and conduct action potentials in neurons (Hodgkin and Huxley, 1952). Nine subtypes of mammalian VGSCs (Nav1.1 to 1.9) have been identified (Noda et al., 1986; Catterall et al., 2005). Seven subtypes of VGSCs are expressed in dorsal root ganglion (DRG) including tetrodotoxin-sensitive (TTX-S) Nav1.1, 1.2, 1.3, 1.6, and 1.7 and tetrodotoxin-resistant (TTX-R) Nav1.8 and Nav1.9 (Dib-Hajj et al., 2010; Ho and O'Leary, 2011). Compared to the major TTX-R VGSC (Nav1.8), TTX-S VGSCs display a lower activation threshold which facilitates the amplification of subthreshold depolarization for action potential generation (Rush et al., 2007). Among TTX-S VGSCs, Nav1.7 is selectively expressed in the peripheral nervous system, while Nav1.1, 1.2, 1.3, and 1.6 are also expressed in the central nervous system (Sangameswaran et al., 1997; Toledo-Aral et al., 1997). Compared to other TTX-S VGSCs, Nav1.7 features a slow closed-state inactivation property which enables Nav1.7 to efficiently amplify slow receptor potentials for action potential generation at free nerve endings (Cummins et al., 1998). Therefore, Nav1.7 is considered a threshold sodium channel in nociceptive DRG neurons and is so-called “peripheral gatekeeper” of pain (Dib-Hajj et al., 2013).

Human mutations of Nav1.7 are associated with multiple inherited pain dysfunction. Loss-of-function mutations of Nav1.7 are associated with congenital insensitivity to pain (CIP) (Cox et al., 2006). In contrast, gain-of-function mutations of Nav1.7 are associated with inherited pain syndromes including inherited erythromelalgia (IEM) (Yang et al., 2004, 2016; Waxman, 2013), paroxysmal extreme pain disorder (PEPD) (Fertleman et al., 2006; Jarecki et al., 2008), and idiopathic small fiber neuropathy (SFN) (Faber et al., 2012).

Increased expression of Nav1.7 has been reported in multiple animal models of pathological pain (Hong et al., 2004; Due et al., 2014; Lee et al., 2014; Emery et al., 2016; Xia et al., 2016; Li et al., 2018). However, it is not clear whether or how much functional Nav1.7 currents are changed in these pain models (Ho and O'Leary, 2011). Although overall biophysical properties are similar among TTX-S isoforms of VGSCs in DRG neurons, Nav1.7 reprimes slower than other isoforms (Herzog et al., 2003). In the current study, we aimed to electrophysiologically dissect subtypes of the TTX-S currents in small DRG neurons of both rats and mice, to subgroup small DRG neurons based on their expression pattern of TTX-S currents, to examine dissected components of TTX-S currents under physiological, pharmacological, pathological, and simulation conditions.

## Experimental procedures

### Cell culture

DRG neurons of adult rats or mice were dissociated and cultured as previously described (Cummins et al., 1998; Tan et al., 2014). Animal procedures were approved by the Indiana University School of Medicine Institutional Animal Care and Use Committee. Briefly, male Sprague Dawley rats (120–150 g, Harlan, Indianapolis, Indiana, United States) or male C57BL/6 mice (20-30 g, Jackson Lab, Bar Harbor, ME, United States) were rendered unconscious by exposure to CO_2_ and decapitated. The lumbar DRGs were excised and then incubated in DMEM (Fisher Scientific, Pittsburgh, PA, United States), containing type 1 collagenase (1.6 mg/mL, Fisher Scientific) and protease (1 mg/mL, Fisher Scientific). After incubation for 30–35 min in a 37°C thermo rocker, the ganglia were sequentially triturated in DMEM supplemented with 10% FBS (Fisher Scientific) and plated on glass coverslips coated with poly-D-lysine (Sigma-Aldrich, St. Louis, Missouri, United States) and laminin (Sigma-Aldrich). Cultures were maintained at 37°C in a humidified 95% air and 5% CO_2_ incubator.

### Electrophysiology

Whole-cell patch-clamp recordings were conducted in DRG neurons of adult rats or mice as previously described (Cummins et al., 1998; Tan et al., 2014; Su et al., 2020). Recordings were obtained from DRG neurons 14–28 h after dissociation. Whole-cell voltage-clamping was applied at room temperature (approximately 22°C) using a HEKA EPC-10 amplifier (HEKA Elektronik, Lambrecht, Germany) or an Axopatch 200B patch-clamp amplifier (Molecular Devices Corporation, Sunnyvale, CA, United States). Data were acquired using the Pulse program (version 8.80; HEKA Elektronik) or pCLAMP 8.0 software (Molecular Devices). Fire-polished electrodes (0.7–1.2 MΩ) were fabricated from 1.7 mm capillary glass using a Sutter P-97 puller (Novato, CA), and the tips were coated with sticky wax (KerrLab) to minimize capacitive artifacts and enable increased series resistance compensation. The standard intracellular solution consisted of 140 mM CsF, 10 mM NaCl, 1.1 mM EGTA, 10 mM HEPES, and pH 7.3. The standard extracellular solution contained 130 mM NaCl, 30 mM TEA chloride, 1 mM MgCl2, 3 mM KCl, 1 mM CaCl_2_, 0.05 mM CdCl_2_, 10 mM HEPES, 10 mM D-glucose, and pH 7.3. Recording solutions were adjusted using D-glucose to maintain physiological osmolality values (305 and 315 Osm for intracellular and extracellular solutions, respectively). All the chemicals were purchased from Sigma-Aldrich except for otherwise mentioned.

Cells on glass coverslips were transferred to a recording chamber containing the 250 μL bathing solution. For neurons subject to IB4 staining, 10 μg/mL IB4-FITC was used for 10 min followed by 2 min rinse (Breese et al., 2005; Wu et al., 2021). Series resistance errors were compensated by 80–90%. Leak currents were linearly canceled by digital P/5 subtraction. Cells were held at a membrane potential of −100 mV. Membrane currents were sampled at 20 kHz and were filtered at 5 kHz. Whole-cell currents were not recorded before 3 min after whole-cell configuration had been established to allow adequate time for the intracellular solution and cytoplasmic milieu to equilibrate. In addition, repetitive depolarization protocols (a series of 60 pulses of 20 ms each to −10 mV) were used to facilitate rundown of Nav1.9-like current. Repriming currents were assayed with a paired depolarization protocol in which the first pulse depolarized the membrane to -20 mV for 20 ms to activate and then inactivate sodium currents, and then it restored the membrane to -100 mV for a period of time ranging from 2 ms to 258 ms before the second pulse depolarizing the membrane to -20 mV for 20 ms again to test the recovered sodium currents.

**Figure 1:**
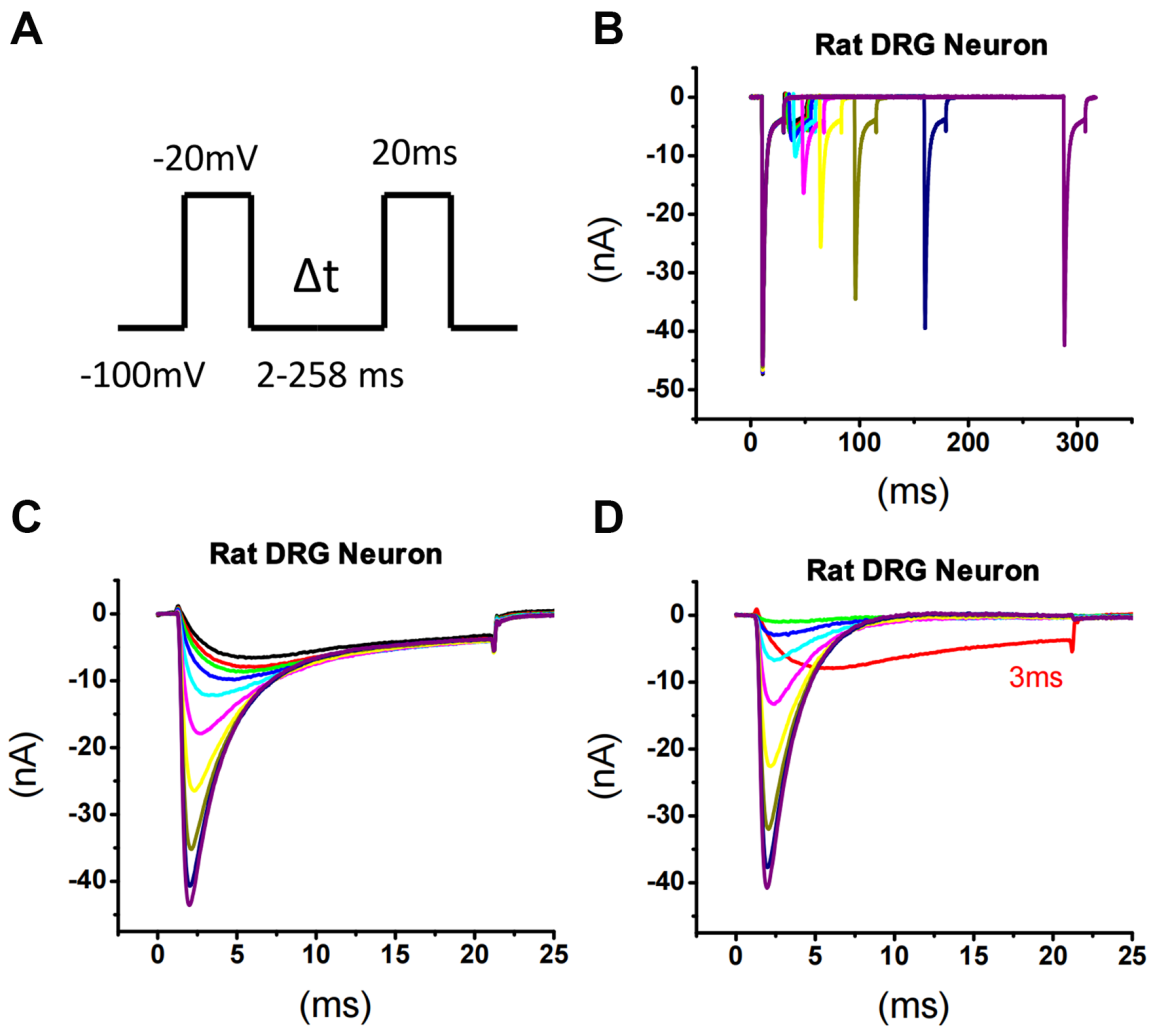
Recording of sodium current repriming in small DRG neurons of rats. **(A)** The voltage protocol used for recording of sodium current repriming. **(B)** Representative traces showing the repriming of sodium currents recorded in a small DRG neuron dissociated from adult Sprague–Dawley rats. **(C)** Comparison of time-dependent repriming of sodium current recorded in A. Note there was a selective increase in fast-activating and fast-inactivating currents after 3 ms repriming (the red trace). **(D)** Separation of TTX-S current from TTX-R current by subtracting maximally recovered TTX-R currents from total sodium currents.

The kinetics of recovered sodium currents were compared. The maximally recovered TTX-R currents were subtracted from the total sodium currents that included both slow activating and inactivating TTX-R currents and fast activating and inactivating TTX-S currents (Figure 1). The subtracted TTX-S currents were plotted against their repriming time interval. The repriming processes were fitted by a double exponential function, and two time constants were obtained for the two terms of exponential fitting. Neurons with identical time constants were defined as single repriming which can be confirmed by fitting with the single exponential function. Time constants larger than 100 ms, which consisted of <5% of the total time constants, were excluded from the current study because those time constants may represent the recovery of sodium currents from slow to intermediate inactivation but not recovering from the fast inactivation of sodium channels which was concerned in this study.

Peak steady-state TTX-S and TTX-R currents were separated by subtracting the maximal steady-state inactivation current of TTX-R from the maximal steady-state inactivation current of the total currents recorded by depolarization to 0 mV from a 500 ms pre-holding at -110 mV (Cummins et al., 1998).

### Neuronal modeling

The membrane excitability of simulated small mouse DRG neurons was studied using the NEURON simulation program (version 7.3) (Hines and Carnevale, 1997). Based on the experimental data recorded from 18 small mouse DRG neurons using whole-cell current clamping, a single-compartment cylindrical model of length 20 μm and radius 20 μm was created to simulate a mouse DRG neuron with 16 pF of whole-cell capacitance. The integration method was Crank–Nicholson at an integration time step (dt) of 0.025 ms.

Simulations were performed assuming room temperature in which the experimental data were recorded. Free ionic concentrations of sodium [(Na^+^)_o_ = 145 mM; (Na^+^)_i_ = 5 mM] and potassium [(K^+^)_o_ = 3 mM; (K^+^)_i_ = 135 mM] were used to calculate their Nernst reversal potential of +86.5 mV (E_Na_) and − 97.7 mV (E_k_), respectively. The linear leakage current was defined as I_Leak_ = g_Leak_(V − E_Leak_), where g_Leak_ is the leak conductance, V is the membrane potential, and E_Leak_ is the reversal potential for the leak current. E_Leak_ was set at −54.9 mV which was also the averaged value of resting membrane potential recorded. g_Leak_ was set at 0.000055 S/cm^2^ which corresponded to the average value of input resistance of 1448MΩ.

The model of small mouse DRG neurons included a leak conductance, two potassium conductance, and two or three sodium conductance (TTX-S Nav1.6 and/or Nav1.7 and TTX-R Nav1.8). The mouse Nav1.6 and Nav1.7 conductance was obtained from DRG neurons of Nav1.8-knockout mice transfected with TTX-R form of mouse Nav1.6 and Nav1.7 channels (Herzog et al., 2003). The Nav1.8 conductance was slightly modified, based on the properties of TTX-R currents recorded in the current study, from a rat Nav1.8 conductance (Sheets et al., 2007). A transient and a delayed rectifier potassium conductance was included (Sheets et al., 2007).

### Statistical analysis

Averaged data were presented as mean ± SEM. The Student *t* test, chi-square test, or one-way ANOVA was used to examine the statistical significance. The significance level was set at a value of p of 0.05.

## Results

### Dissecting slow and fast-repriming components of TTX-S currents in small DRG neurons of rats

Sodium currents were recorded in small-sized DRG neurons (D < 30 μm) dissociated from adult rats. To separate the different components of TTX-S current, a repriming protocol (Figure 1A) was designed to first separate TTX-S from TTX-R currents and then to separate the slow-repriming (SR) from the fast-repriming (FR) component of TTX-S currents. To separate the TTX-S from TTX-R currents, a test pulse of -20 mV was chosen for sodium current activation. At -20 mV, approximately 80% TTX-S currents were activated, while less than 40% TTX-R currents were activated (Rush et al., 2007). In addition to differential current amplitude activated by -20 mV test pulse, the activation time constant of TTX-R currents also peaked at -20 mV (TTX-R currents activate slowest at this voltage) (Sheets et al., 2007). Therefore, both a small amount of activation and the slowest activation kinetics of TTX-R currents were chosen in this protocol. To separate the SR and FR components of TTX-S currents, -100 mV was chosen as the holding and repriming voltage. This holding/repriming voltage allowed a good difference in repriming time constants between fast and slow components (compared to more negative voltages) and prevented a too slow time constant for the SR component (compared to more positive voltages) (Herzog et al., 2003) which could overlap with the repriming of slow/intermediate inactivation of sodium channels. Therefore, the protocol used here utilized the differential properties both between TTX-S and TTX-R currents and between SR and FR components of TTX-S currents.

As shown in Figure 1, sodium currents recovered as the repriming intervals increased (Figure 1B). By comparing the currents recovering over time (Figure 1C), it was found that current amplitude increased with a change in current kinetics from 3 ms (red trace) on. From 2 ms to 3 ms, the current amplitude slightly increased without an apparent change in the overall current kinetics. From 3 ms on, the increase in currents was associated with a leftward shift in time-to-peak. Moreover, the increases happened during the rising and fast-inactivating phases of currents, but to the least, in the slow-inactivating phase. These changes suggested that there was a fast-activating and fast-inactivating current component selectively recovered from 3 ms on. As it is known that TTX-S currents are the fast-activating and fast-inactivating sodium currents expressed in DRG neurons, these results suggested that TTX-S currents started to recover from 3 ms on. Therefore, TTX-R currents were subtracted from total sodium currents resulting in the separation of TTX-S currents repriming over time (Figure 1D).

**Figure 2:**
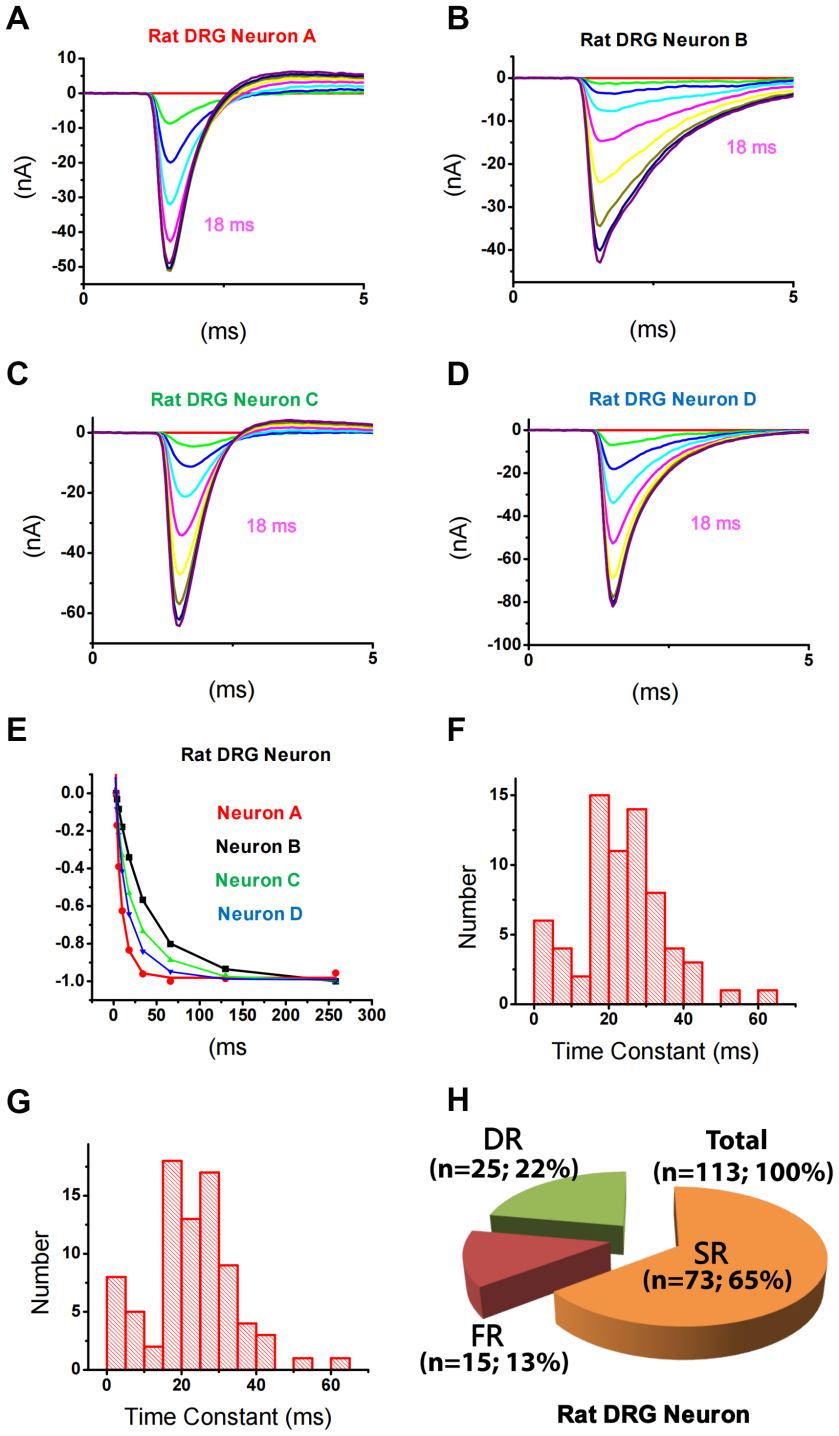
Differential repriming of TTX-S currents in small DRG neurons of rats. **(A–D)** Representative traces showing the repriming of TTX-S sodium currents recorded from four small DRG neurons using the protocol presented in Figure 1. **(E)** The amplitude of each current was normalized to the current recorded at 258 ms in each neuron as shown in **A–D**. The current in **A** (red) and **B** (black) were best fitted by a single exponential function, while the current in **C** (blue) and **D** (green) were best fitted by a double exponential function. **(F)** The distribution of time constant for single-repriming TTX-S currents. **(G)** The distribution of combined single-repriming time constants and the faster time constants in neurons that had a slower time constant larger than 70 ms. The distribution pattern of fast and slow time constant domains is similar between **(F,G)**. The two time constants fell in the 10–15 ms range were 10.1 and 11.2 ms. **(H)** DRG neurons were divided into three different groups: slow-repriming (SR), fast-repriming (FR), and double-repriming (DR) neurons based on their components of repriming TTX-S current, respectively.

Using this strategy, TTX-S currents in 113 small DRG neurons were separated. It was noticed that repriming processes for TTX-S currents were different among neurons. For example, at a repriming interval of 18 ms (purple trace), TTX-S currents in neuron A (Figure 2A) almost fully recovered, while only a small portion of currents in neuron B (Figure 2B) recovered. To examine the fast and slow components of repriming currents, repriming processes were fitted by the double exponential function. Some neurons were best fitted by the double exponential function (Figures 2C−E; green/triangle up and blue/triangle down), while others were best fitted by the single exponential function (Figures 2A,B,E; black/square and red/circle). To understand the distribution of repriming time constant for single-repriming cells (whose repriming processes were best fitted by the single exponential function; *n* = 69), a histogram graph (Figure 2F) was plotted to show the frequency count of repriming time constants in those cells. All the time constants were between 0 and 70 ms, and there were both SR (>15 ms) and FR (<12 ms) components. It was further included in Figure 2G with a time constant of <70 ms from cells (whose repriming processes were best fitted by double exponential functions; *n* = 12) that had another time constant >70 ms. A similar distribution pattern was observed in Figure 2G compared to Figure 2F. It was indicated that time constants larger than 70 ms might fall out of the repriming process for fast inactivation, which was the interest of the current study, and fall into the repriming process for slow/intermediate inactivation. Therefore, the neurons with one of the two time constant larger than 70 ms were treated as single-repriming cells. For neurons with two time constant in the range of 0–70 ms, it was found that the majority of them (25 out of 32) had both SR and FR components. The largest time constant for the FR component was 12.0 ms, while the smallest time constant for the SR component was 18.4 ms. Other seven neurons had both time constants between 15 and 70 ms. Because there was no FR component, these seven neurons were treated as SR neurons.

### Expression pattern of slow and fast-repriming TTX-S currents in small DRG neurons of rats

Based on the above-mentioned analysis, small DRG neurons dissociated from rats were divided into three groups (Figure 2H): (1) SR neurons that had a SR time constant in the 15–70 ms range without a FR time constant in the 0–12 ms range; (2) FR neurons that had a FR time constant in the 0–12 ms range without a SR time constant in the 15–70 ms range; and (3) double-repriming (DR) neurons that had both a FR time constant in the 0–12 ms range and a SR time constant in the 15–70 ms range. Among 113 neurons studied, there were 73, 15, and 25 neurons in SR, FR, and DR neuron groups, respectively.

The cellular electrophysiological properties among these three groups of neurons were compared. It was found that there were no significant differences in membrane capacitance and current density of TTX-S and TTX-R currents (Table 1, One-Way ANOVA). TTX-S and TTX-R currents were separated by subtracting the maximal steady-state inactivation current of TTX-R from the maximal steady-state inactivation current of the total currents recorded by depolarization to 0 mV from a 500 ms pre-holding at -110 mV (Cummins et al., 1998).

**Table 1:** Comparison of sodium currents among subpopulations of small DRG neurons and between the rat and mouse.

	Rat			Mouse		
Subpopulation	SR	DR	FR	SR	DR	FR
*N*	73	25	15	2	60	15
Cm (pF)	26.1 ± 1.1	25.0 ± 1.7	27.1 ± 1.5	16.6	14.8 ± 1.7	14.6 ± 0.5
*I*_TTX-S_ (nA/pF)	1.64 ± 0.08	1.34 ± 0.14	1.33 ± 0.30	1.21	2.19 ± 0.13	2.10 ± 0.21
*I*_TTX-R_ (nA/pF)	1.25 ± 0.06	1.09 ± 0.12	0.92 ± 0.21	1.36	0.51 ± 0.05	0.59 ± 0.15
*I*_SR_ (nA/pF)	1.85 ± 0.20	0.87 ± 0.08		1.96	1.23 ± 0.08	
*I*_FR_ (nA/pF)		1.07 ± 0.08	2.27 ± 0.24		1.45 ± 0.08	2.54 ± 0.12

SR, slow-repriming neurons; DR, double-repriming neurons; FR, fast-repriming neurons.

### Dissecting and expression pattern of slow and fast-repriming TTX-S currents in small DRG neurons of mice

**Figure 3:**
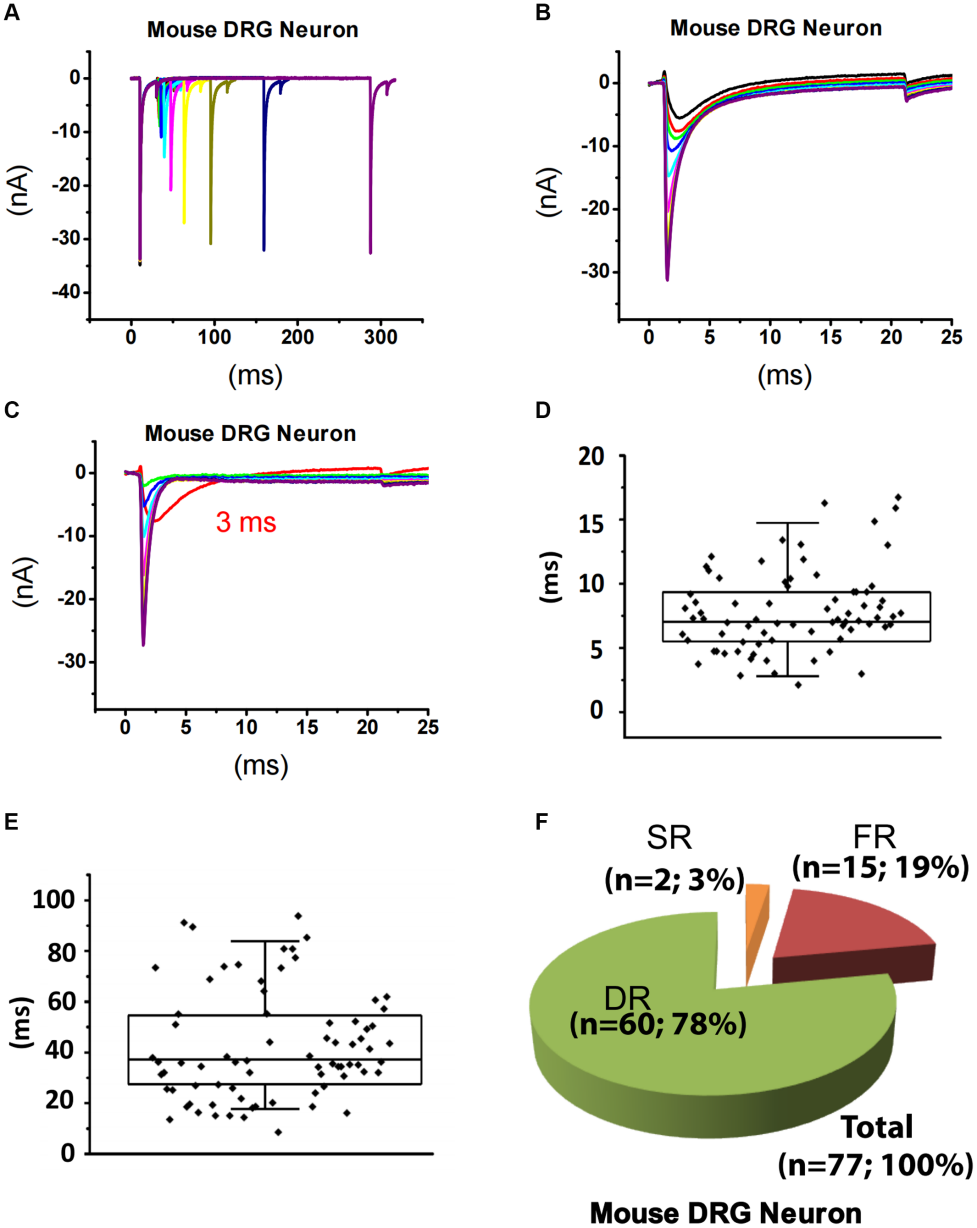
Recording of sodium current repriming in small DRG neurons of mice. **(A)** Representative traces showing the repriming of sodium currents recorded in a small DRG neuron dissociated from adult C57BL/6 mice. The voltage protocol used for recording of sodium current repriming is the same as shown in Figure 1. **(B)** Comparison of time-dependent repriming of sodium current recorded in A. Note there was a selective increase in fast-activating and fast-inactivating currents after 3 ms repriming (the red trace). **(C)** Separation of TTX-S current from TTX-R current by subtracting maximally recovered TTX-R currents from total sodium currents. The box plots of the distribution of the fast **(D)** and the slow **(E)** time constants. **(F)** DRG neurons were divided into three different groups: slow-repriming (SR), fast-repriming (FR), and double-repriming (DR) neurons based on their components of repriming TTX-S currents, respectively.

Using the same strategy for rat DRG neurons, TTX-S currents of 84 small DRG neurons (D < 25 μm) dissociated from adult mice were separated from TTX-R currents (Figures 3A–C). After fitting the repriming process of TTX-S currents, it was found that a majority (approximately 80%) of the mouse neurons were best fitted by two exponential functions. To determine the time constant range of fast and slow components in small-sized mouse DRG neurons, box plots of the faster and the slower time constants were drawn for neurons best fitted by two exponential functions, respectively (Figures 3D,E). For distribution of the faster time constant, a maximal value of 14.8 ms was found and time constants larger than 14.8 ms were excluded from this group as outliners (Figure 3D). For distribution of the slower time constant, a minimum of 17.7 ms and a maximum of 84.5 ms was found (Figure 3E). Taken together, time constants less than 14.8 ms were considered as fast that corresponds to FR current, while time constants between 17.7 and 84.5 ms were considered as slow related to SR current. On the other hand, time constants between 14.8 and 17.7 ms were not considered as either fast or slow, while time constants over 84.5 ms were considered for slow/intermediate inactivation.

Based on the above criteria, small DRG neurons dissociated from mice were divided into three groups: (1) SR neurons that had a SR time constant in the 17.7–84.5 ms range without a FR time constant in the 0–14.8 ms range; (2) FR neurons that had a FR time constant in the 0–14.8 ms range without a SR time constant in the 17.7–84.5 ms range; and (3) DR neurons that had both a FR time constant in the 0–14.8 ms range and a SR time constant in the 17.7–84.5 ms range. Among 84 neurons studied, there are 2, 15, and 60 neurons in the SR, FR, and DR neuron groups, respectively (Figure 3F). Neurons with a time constant between 14.8 and 17.7 ms (*n* = 7) were excluded from classification.

The cellular electrophysiological properties among these three groups of neurons were compared in Table 1. The properties of the SR neurons were not compared to the other two groups due to their small number of 2. The membrane capacitance and current density of TTX-S and TTX-R currents were not different between the DR and FR groups (Table 1).

### Effects of ProTx-II on slow and fast-repriming TTX-S currents in small DRG neurons

**Figure 4:**
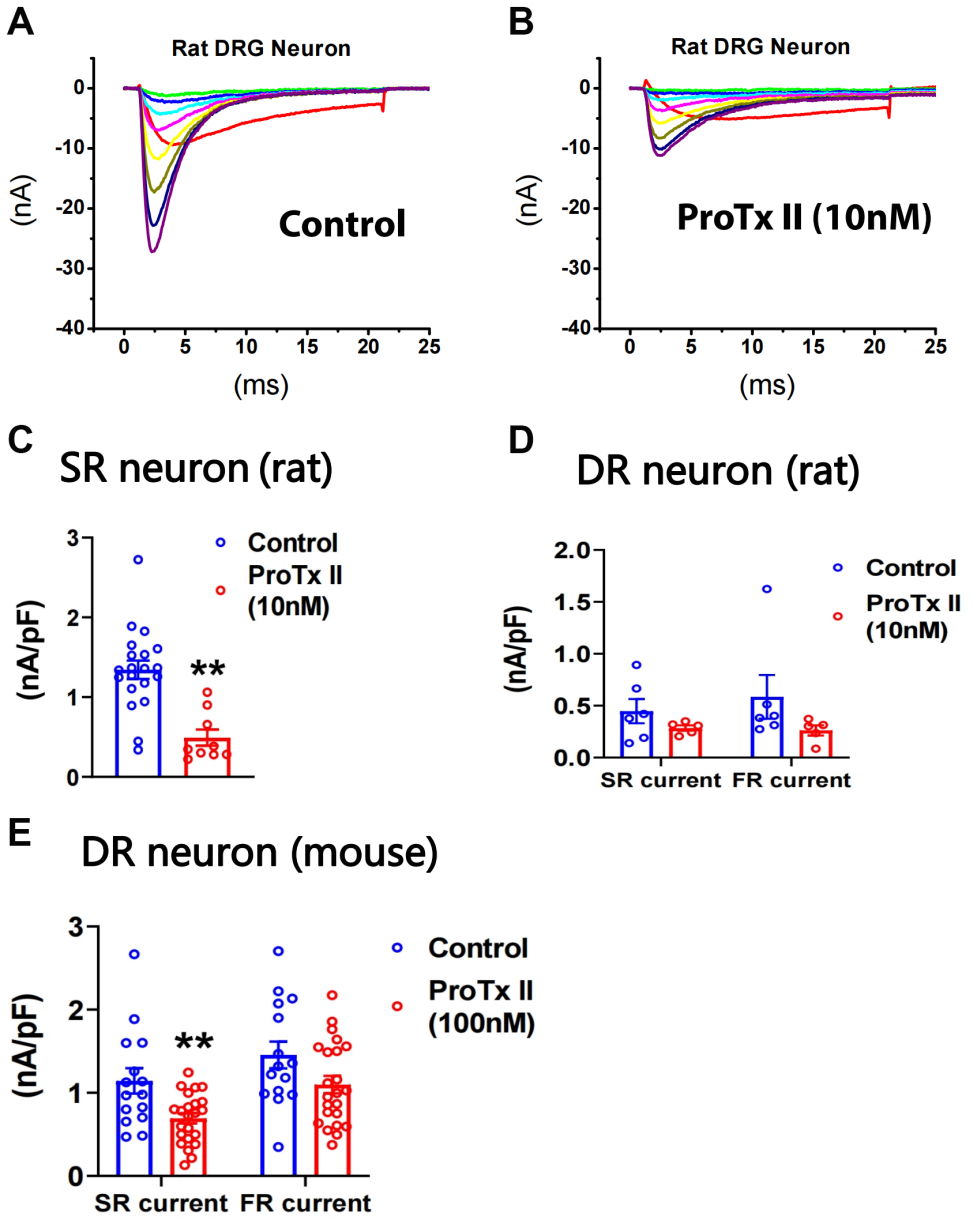
Effects of ProTx-II on repriming of sodium currents in small DRG neurons. ProTx-II was pre-treated in culture plates for 45 min and kept in a recording chamber for 10–90 min before the repriming of sodium currents was recorded in small DRG neurons. Representative traces of sodium currents repriming in rat neurons were shown in control **(A)** and ProTx-II **(B)** groups. ProTx-II did not inhibit TTX-R currents in either rat or mouse neurons (see Results). However, it significantly inhibited SR currents in SR rat neurons **(C)** and the SR currents in DR mouse neurons **(E)**. In addition, ProTx-II did not significantly decrease both SR and FR currents in DR rat neurons **(D)** or in DR mouse neurons **(E)**. Data were presented as mean ± standard errors of mean. **, *p* < 0.01, Student *t*-test.

It has been reported that ProTx-II selectively inhibits Nav1.7 *in vitro* (Schmalhofer et al., 2008). We examined the effects of ProTx-II on SR and FR components of TTX-S currents in small DRG neurons of both rats and mice. In rat neurons, pre-treatment of 10 nM ProTx-II did not significantly inhibit TTX-R currents (0.38 ± 0.06 and 0.32 ± 0.07 nA/pF for control (*n* = 26) and ProTx-II (*n* = 14), respectively; Figures 4A,B). However, 10 nM ProTx-II significantly inhibited SR currents in SR cells (Figure 4C) but not SR or FR currents in DR neurons (Figure 4D). The effects of ProTx-II on FR neurons were not compared due to their small number. In mouse neurons, pre-treatment of 100 nM ProTx-II significantly inhibited SR currents in DR cells (Figure 4E). However, 30 nM ProTx-II did not inhibit either SR or FR currents (1.29 ± 0.24 and 1.07 ± 0.28 nA/pF and 0.96 ± 0.10 and 0.91 ± 0.33 nA/pF for fast_control (*n* = 11) and fast_ProTx-II (*n* = 8) and for slow_control (*n* = 11) and slow_ProTx-II (*n* = 8), respectively). In the study, 100 nM ProTx-II did not inhibit TTX-R currents (0.40 ± 0.03 and 0.42 ± 0.05 nA/pF for control (*n* = 17) and ProTx-II (*n* = 26), respectively).

**Figure 5:**
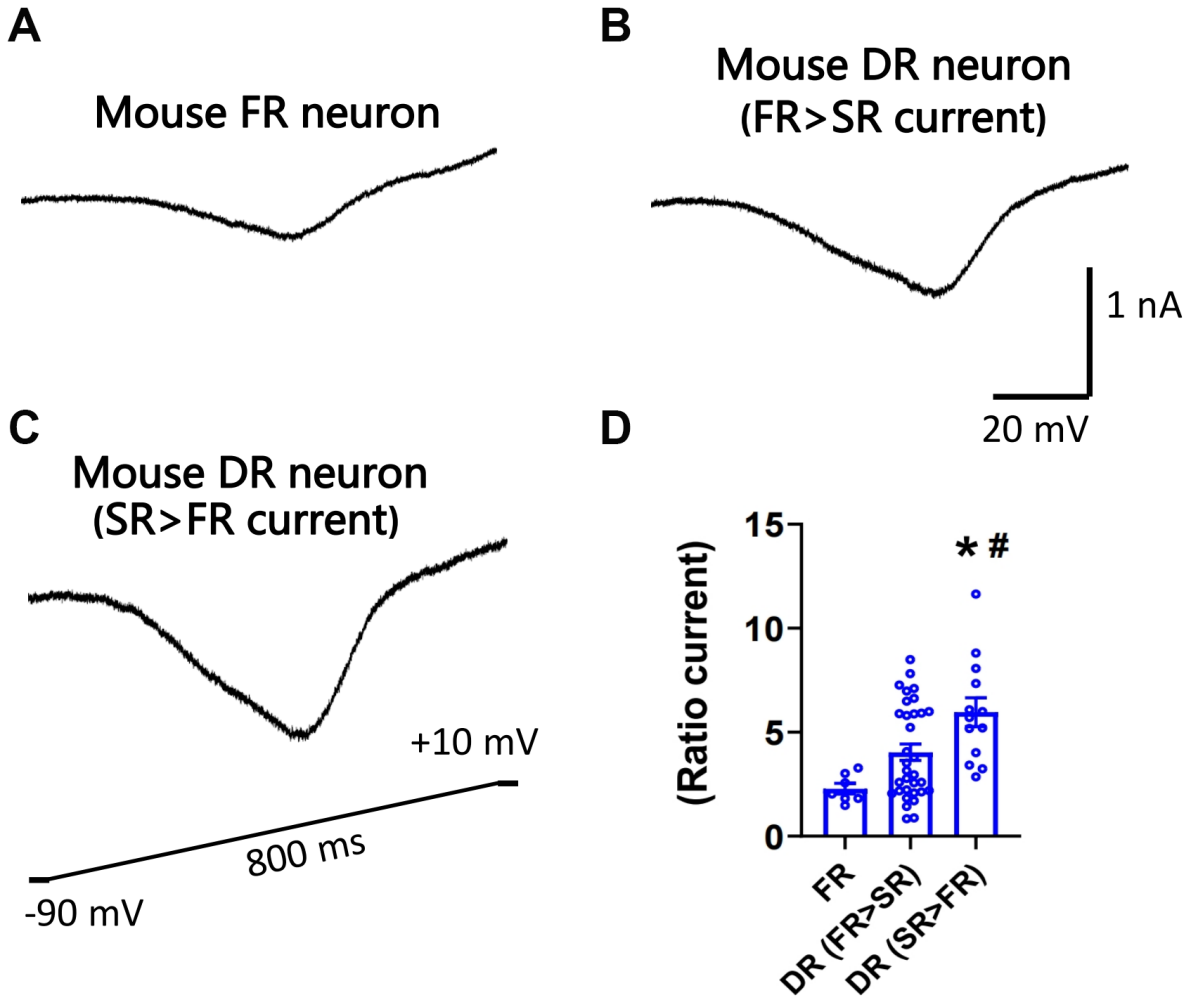
Recording of slow ramp currents in small DRG neurons of mice. Ramp sodium currents were activated by a slow ramp voltage protocol (−90 to +10 mV in 800 ms) in small DRG neurons dissociated from adult C57BL/6 mice. Five voltage ramps were applied to each neuron, and the five ramp currents were averaged. Representative traces of ramp currents were recorded from an FR neuron **(A)**, a DR neuron with higher FR than SR currents **(B)**, and a DR neuron with higher SR than FR currents **(C)**. **(D)** The slow ramp currents were significantly different among the three groups of neurons (*p* < 0.01, one-way ANOVA). Data were presented as mean ± standard errors of mean. *, *p* < 0.01 (vs. FR neuron), post-hoc Tukey test; #, *p* < 0.05 (vs. DR neuron with higher FR than SR currents), post-hoc Tukey test.

Nav1.7 generates larger slow ramp currents compared to other TTX-S isoforms expressed in DRG neurons. To test whether neurons expressing more SR currents can generate larger slow ramp currents, both repriming currents and slow ramp currents were recorded in the same DRG neurons of mice. Peak ramp currents were normalized to the maximal repriming TTX-S currents. Ratio ramp currents were compared among three groups of neurons: FR neurons, DR neurons with SR current amplitude < FR current amplitude, and DR neurons with SR current amplitude > FR current amplitude. No SR neurons were studied due to their small percentage. As shown in Figure 5, ratio ramp currents were significantly different among the three groups of neurons (Figure 5D). Ratio ramp currents in the DR neurons with more SR currents (than FR currents) were significantly larger than those in the other two groups (Figure 5D).

### Effects of paclitaxel and high glucose on the expression of slow and fast-repriming TTX-S currents in IB_4_^+^ and IB_4_^−^ small DRG neurons

**Figure 6:**
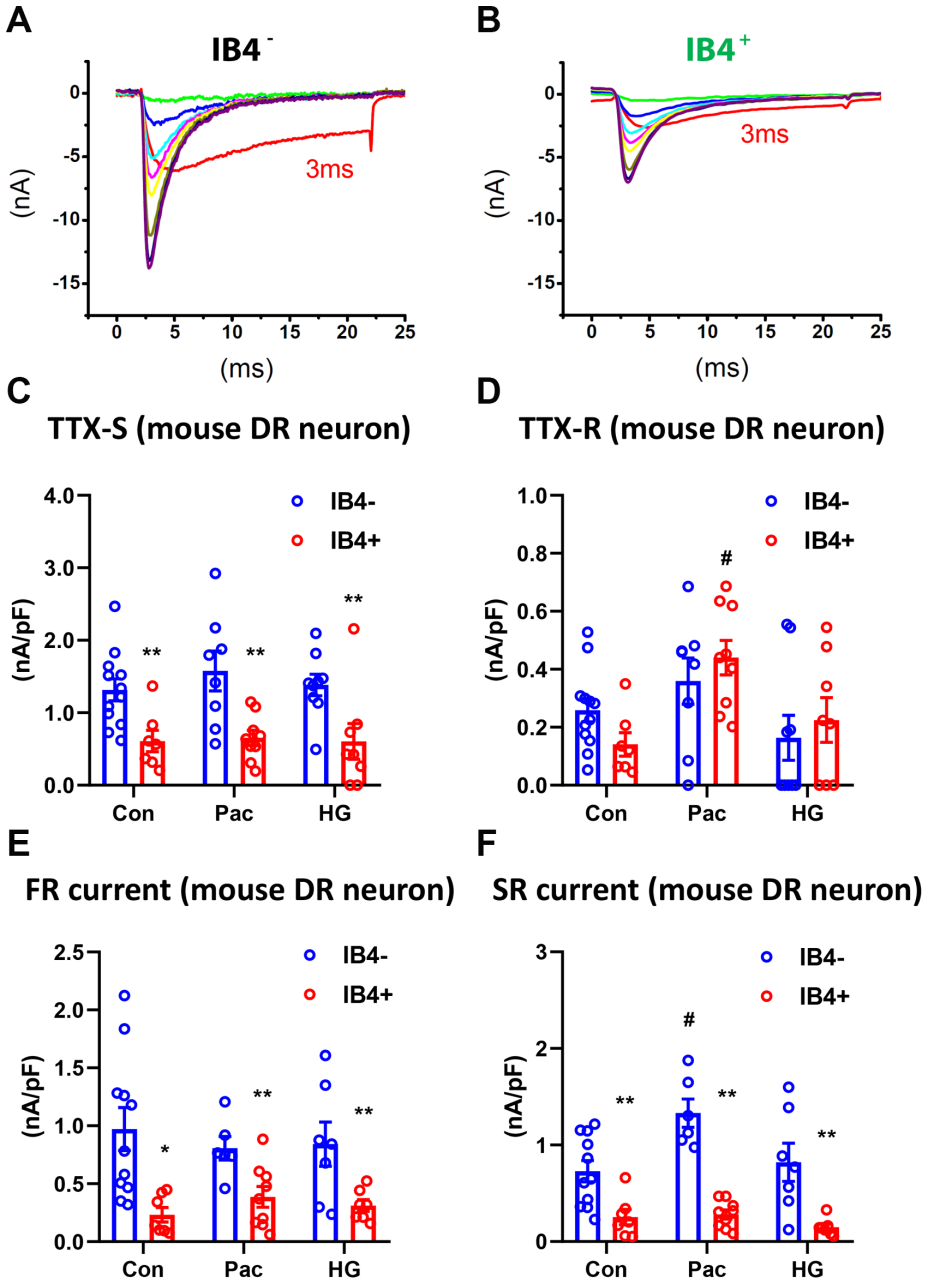
Recording of fast and slow-repriming TTX-S currents in small DRG neurons of mice. Representative traces showing the dissection of repriming TTX-S currents recorded in a IB_4_^+^ **(A)** and a IB_4_^−^ **(B)** small DRG neuron dissociated from adult C57BL/6 mice. The voltage protocol used for recording of sodium current repriming is the same as shown in Figure 1. Current density of total TTX-S **(C)**, TTX-R **(D)**, SR TTX-S currents **(E)**, and FR TTX-S currents **(F)** in IB_4_^+^ (green bars) and IB_4_^−^ (gray bars) neurons were compared among control (Con), paclitaxel (Pac), and high glucose (HG) groups. Paclitaxel (1 μM) or high glucose (30 mM compared to 10 mM in control) were pre-treated overnight in the culture medium. Data were presented as mean ± standard errors of mean. **, *p* < 0.01 (vs. IB_4_^−^ neurons), student *t* test; #, *p* < 0.05 (vs. control), one-way ANOVA and post-hoc Tukey test.

To study the functional implication of SR and FR TTX-S currents, we examined their expression in IB_4_^+^ and IB_4_^−^ subpopulations of small mouse DRG neurons in the absence or presence of paclitaxel or high glucose (Chattopadhyay et al., 2008; Chang et al., 2017). In the control IB_4_^+^ group, there were 1 SR, 5 FR, and 11 DR neurons. In the control IB_4_^−^ group, there were 1 SR, 1 FR, and 12 DR neurons. Compared to IB_4_^−^ neurons, there was a significantly higher percentage of FR neurons in IB_4_^+^ neurons (29% vs. 7%, chi square test). However, the density of TTX-S currents, but not TTX-R currents, was significantly higher in the IB_4_^−^ group compared to the IB_4_^+^ group (Figures 6A–D). Moreover, the density of SR and FR TTX-S currents in DR neurons was significantly higher in the IB_4_^−^ group compared to the IB_4_^+^ group (Figures 6E,F). The current density was not compared in SR or FR neuron groups due to the limited number of recordings.

Overnight pre-treatment of paclitaxel or high glucose increases expression of Nav1.7 (Chattopadhyay et al., 2008, Chang et al., 2017). We compared the SR and FR TTX-S currents of DR neurons in both IB_4_^+^ and IB_4_^−^ subpopulations of small mouse DRG neurons in the absence or presence of overnight pre-treatment of paclitaxel (1 μM) or high glucose (30 mM compared to 10 mM in control). Compared to the control, paclitaxel selectively increased SR but not FR TTX-S currents in IB_4_^−^ neurons (one-way ANOVA, Figures 6E,F). Paclitaxel did not change FR currents in IB_4_^−^ neurons or either component of TTX-S currents in IB_4_^+^ neurons. On the other hand, high glucose did not change either SR or FR TTX-S currents in either IB_4_^−^ or IB_4_^+^ neurons. In addition to the SR TTX-S currents, paclitaxel also significantly increased the current density of the TTX-R currents (one-way ANOVA, Figure 6D).

### Contribution of slow and fast-repriming TTX-S currents to membrane excitability in small DRG neuron models

**Figure 7:**
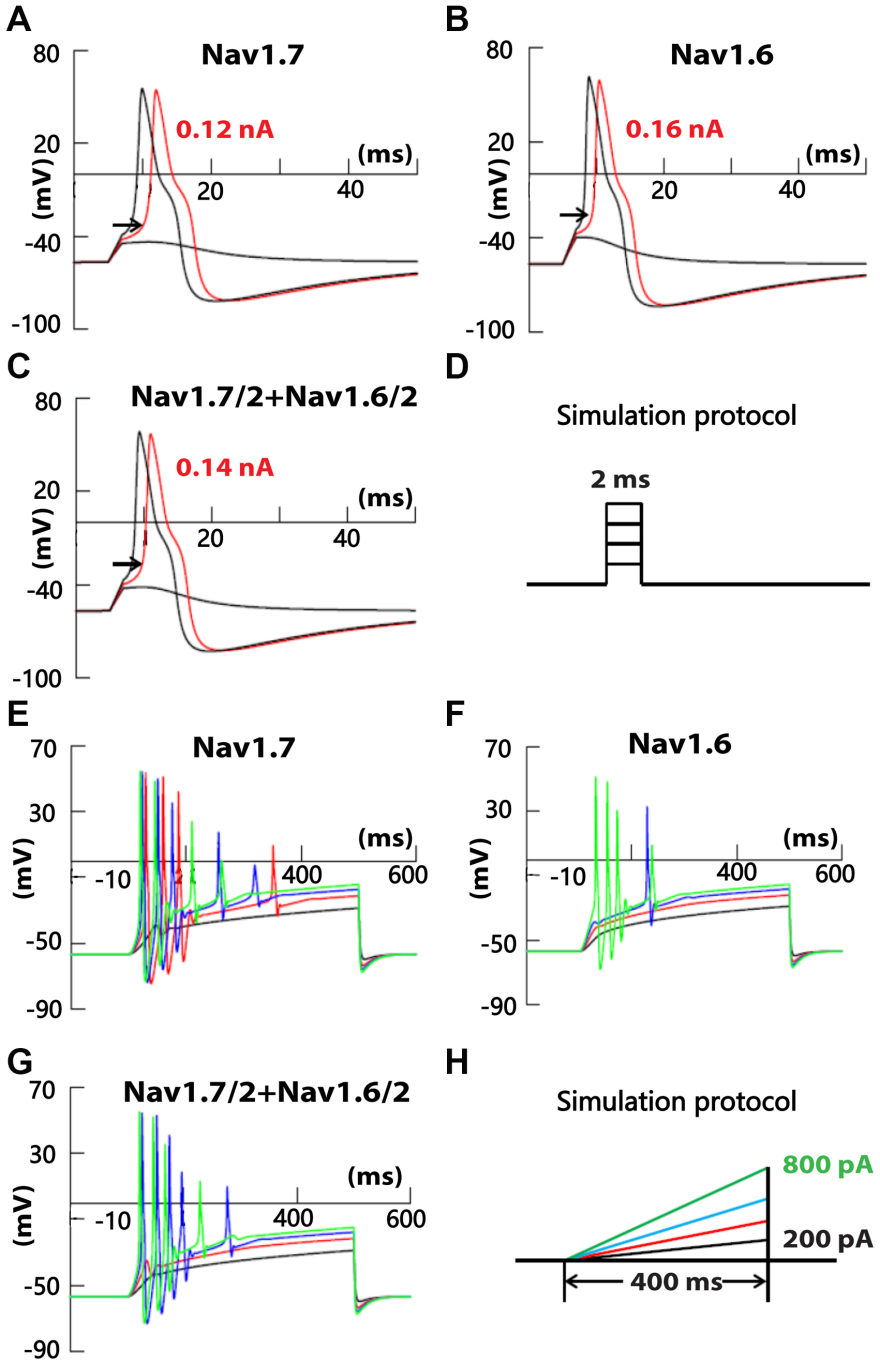
Recording of membrane excitability in simulated small DRG neurons of mice. A cell model of a small mouse DRG neuron was constructed using NEURON simulation software. Mouse Nav1.6 and/or Nav1.7 channels were inserted into the model cell, along with TTX-R Nav1.8, transient and delayed rectifier potassium channels. Action potentials were triggered by a series of square **(D)** or ramp **(H)** current injection in model neurons with Nav1.7 but not Nav1.6 **(A,E)**, half amount of Nav1.7 and Nav1.6 **(B,F)**, and Nav1.6 but not Nav1.7 **(C,G)**, respectively. The amount of Nav1.6 in Nav1.6 neurons was the same as the amount of Nav1.7 in Nav1.7 neurons.

To test the possible differential contribution of SR and FR TTX-S currents to membrane excitability of small DRG neurons, simulation experiments were conducted on a DRG neuron model. A cell model of small-sized mouse DRG neurons was constructed based on the whole-cell current clamp recording of 18 small DRG neurons of adult C57BL/6 mice. TTX-S and fast-repriming Nav1.6, TTX-S and slow-repriming Nav1.7, TTX-R Nav1.8, transient and sustained potassium channels were incorporated into the model neuron. To test the contribution of fast-repriming Nav1.6 and slow-repriming Nav1.7 to the membrane excitability, three types of model neurons were constructed: the Nav1.6 neuron with Nav1.6 but not Nav1.7, the Nav1.7 neuron with Nav1.7 but not Nav1.6, and the Nav1.6/1.7 neuron with half amount of Nav1.6 and Nav1.7. The amount of Nav1.6 in the Nav1.6 neuron was the same as the amount of Nav1.7 in the Nav1.7 neuron. As shown in Figures 7A–D, action potentials were triggered by a series of 2 ms square current injections. The rheobase of action potent was the lowest in the Nav1.7 neuron, while it was the highest in the Nav1.6 neuron. Similarly, the voltage threshold of action potential was the lowest in the Nav1.7 neuron (−32.5 mV), while it was the highest in the Nav1.6 neuron (−26.5 mV). Both the rheobase and voltage threshold (−29.0 mV) of action potential in the Nav1.6/1.7 neuron were in between those of the other two groups. As Nav1.7 generates larger ramp currents compared to Nav1.6, a series of ramp current were injected into the three types of neurons (Figures 7E−H). In the Nav1.7 neuron, four, five, and four action potentials were generated by ramp current injection at 400, 600, and 800 pA, respectively (Figure 7E). The same current injections only generated zero, one, and four action potentials in the Nav1.6 neuron (Figure 7F). In the Nav1.6/1.7 neuron, the same series of current injections produced zero, five, and four action potentials (Figure 7G).

## Discussion

In the current study, an SR and an FR component of TTX-S current are electrophysiologically dissected in small-sized DRG neurons of both rats and mice. The time constant of SR TTX-S currents is similar to that of the TTX-R Nav1.7 transfected to DRG neurons dissociated from Nav1.8 knock-out mice (Herzog et al., 2003). Moreover, SR TTX-S current is inhibited by ProTx-II and is associated with larger slow ramp currents compared to FR TTX-S current. Taken together, it is suggested that SR TTX-S current recorded in the current study is mediated by endogenous Nav1.7 expressed in small DRG neurons.

The separation of SR and FR TTX-S currents may serve to validate the efficacy and selectivity of Nav1.7 inhibitors on endogenous Nav1.7 currents in DRG neurons. For example, ProTx-II inhibits Nav1.7 with IC_50_ of 0.3 or 0.7 nM *in vitro* (Schmalhofer et al., 2008; Xiao et al., 2010). However, in the current study, ProTx-II significantly inhibits SR TTX-S currents at concentrations of 10 nM and 100 nM for rat and mouse DRG neurons, respectively. At these concentrations, ProTx-II also insignificantly inhibits FR TTX-S currents. These results suggest that ProTx-II might be less effective and selective in inhibiting endogenous Nav1.7 currents compared to Nav1.7 expressed *in vitro*. Interestingly, it has been reported that Nav1.2 loses its sensitivity to 100 nM ProTx-II when co-expressed with sodium channel β4 subunits (Gilchrist et al., 2013). Since β4 subunits are expressed in DRG neurons (Tan et al., 2014; Xie et al., 2016), it might be suggested that β4 subunits contribute to the lower efficacy and selectivity of ProTx-II, inhibiting endogenous Nav1.7 currents in DRG neurons. PF-05198007 is a potent and selective Nav1.7 inhibitor that blocks a majority of TTX-S currents in small DRG neurons of mice (Alexandrou et al., 2016). Future studies may utilize the dissecting protocol described in the current study, combined with recently developed selective Nav1.8 blockers (Jones et al., 2023), to validate efficacy and selectivity of Nav1.7 inhibitors for endogenous Nav1.7 currents in DRG neurons.

The current study has identified three groups of small DRG neurons based on the expression pattern of SR and FR TTX-S currents in both rats and mice. However, the majority (65%) of rat neurons are SR neurons, while the majority (78%) of mouse neurons are DR neurons. In addition, approximately 20% of rat neurons are DR neurons, while approximately 20% of mouse neurons are FR neurons. These results indicate that expression of Nav1.7 might be much higher and combinational expression of Nav1.7 and FR currents might be much lower in rat small DRG neurons compared to mice. As many studies utilize the mouse model for easier adaptation to transgenic studies (Liu and Wood, 2011) while other studies prefer the rat model for easier surgical procedures (Xie et al., 2016), it may be important to take into consideration the differential expression of functional Nav1.7 in rats and mice. Moreover, the differential expression of Nav1.7 between rats and mice might suggest that Nav1.7 plays a more important role in primary nociception of rats compared to mice. Considering the current failure of the development of Nav1.7 inhibitors into painkillers in clinical trials (Eagles et al., 2022), it might be speculated that in human DRG, Nav1.7 plays a less important role compared to rats and that targeting Nav1.7 alone may not achieve sufficient pain-reducing effects.

Previous studies have found that small IB_4_^−^ neurons express higher TTX-S currents compared to IB_4_^+^ neurons in DRG (Wang et al., 2008). The current study identifies that the majority of either IB_4_^−^ or IB_4_^+^ small neurons are DR neurons in mouse DRG. Moreover, both SR and FR TTX-S current are larger in IB_4_^−^ compared to IB_4_^+^ DR neurons. These results might suggest that a majority of peptidergic DRG neurons (IB_4_^−^) have a lower threshold for action potential generation and a higher intrinsic excitability compared to non-peptidergic DRG neurons (IB_4_^+^). However, our recent current clamp study revealed that mouse IB_4_^+^ neurons display a lower rheobase of action potential compared to IB_4_^−^ neurons (Wu et al., 2021). Nevertheless, this contradiction can be explained by other properties of these neurons. For example, mouse IB_4_^+^ neurons have a more depolarized resting membrane potential compared to IB_4_^−^ neurons (Wu et al., 2021). The depolarized resting membrane potential likely contributes to the lower rheobase of action potential in mouse IB_4_^+^ neurons. On the other hand, mouse IB_4_^−^ neurons generate more action potentials upon supra-threshold current injections compared to IB_4_^+^ neurons (Wu et al., 2021). This difference might involve the higher expression of both SR and FR TTX-S currents, but not TTX-R currents, in IB_4_^−^ neurons compared to IB_4_^+^ neurons. However, differential expression of other ion channels such as voltage-gated potassium and calcium channels in these two groups of neurons could contribute to differential supra-threshold responses as well (Wu and Pan, 2004; Vydyanathan et al., 2005).

Multiple studies have found that paclitaxel increases Nav1.7 in the DRG neuron following *in vivo* treatment (Xia et al., 2016; Li et al., 2018). Moreover, a previous study has found that paclitaxel selectively increases Nav1.7 but not Nav1.8 in DRG neuron culture (Chang et al., 2017). The current study suggests that paclitaxel increases Nav1.7 in IB_4_^−^ neurons selectively. Moreover, paclitaxel does not increase FR TTX-S currents indicating the increasing effects of paclitaxel is selective to Nav1.7 but no other TTX-S sodium channels. However, the current study reveals an increasing effect of paclitaxel on TTX-R currents in IB_4_^+^ neurons, while a previous study did not find an increasing effect of paclitaxel on Nav1.8 (Chang et al., 2017). This might be due to the difference in methods (patch clamp vs. qPCR) or in species (mouse vs. human). In contrast to previous findings that diabetes or high glucose increases Nav1.7 and Nav1.8 in DRG neurons, the current study does not find changes in SR TTX-S current or TTX-R current following high glucose treatment (Hong et al., 2004; Chattopadhyay et al., 2008; Sun et al., 2012; Hu et al., 2016; Nakatani et al., 2020). Multiple factors could contribute to the difference including species, age, *in vivo* or *in vitro* treatment, levels of high glucose (30 vs. 45 mM), and examining methods. Overall, studying SR and FR TTX-S currents in both IB_4_^+^ and IB_4_^−^ neurons might be an efficient way to explore the functional change of VGSCs in DRG neurons.

Although the current study does not use animal models of pain, the separation of SR and FR TTX-S could be applied to chronic pain models. A potential confounding sodium channel isoform in chronic pain seems to be Nav1.3 which is re-expressed in adult DRG neurons following peripheral nerve injuries (Waxman et al., 1994; Cummins and Waxman, 1997; Kim et al., 2002; Hong et al., 2004). However, a TTX-R Nav1.3 expressed in DRG neurons displays a similar repriming time constant at relevant negative membrane potentials (for example 4 ms at -100 mV) compared to a TTX-R Nav1.6 (3 ms at -100 mV; Cummins et al., 2001, Herzog et al., 2003). Nav1.6 is presumably the major FR component recorded in the current study. Moreover, in the large DRG neurons following axotomy (which is supposed to have increased Nav1.3 and decreased Nav1.6), the repriming time constant at -100 mV is indistinguishable between control and axotomy groups (Everill et al., 2001). These findings suggest that Nav1.3 may not significantly confound the separation of SR and FR TTX-S components in DRG neurons following nerve injury. Another potential confounding sodium channel isoform (Nav1.8) is often up-regulated in inflammatory pain models (Tanaka et al., 1998; Yu et al., 2011). Although a couple of studies do not suggest a significant change in Nav1.8 repriming in inflammatory pain models (Bielefeldt et al., 2002; Biet et al., 2021), one study suggests that chronic tumor necrosis factor slows the repriming of Nav1.8 which may confound the separation of TTX-S currents from Nav1.8-mediated TTX-R currents using the current separation protocol (Fischer et al., 2017). Therefore, for pain models that cause slowed repriming of Nav1.8, a selective Nav1.8 blocker (Jones et al., 2023) may be used in combination with the current separation protocol.

Modulation of sodium channels by protein kinases has been well studied, and their effects on channel repriming have not been reported much. A previous study has found that PKA does not affect Nav1.7 repriming (Chatelier et al., 2008). In addition, Nav1.6 (which is presumably the major FS component in the current study) is largely resistant to PKA or PKC (Chen et al., 2008). Based on these findings, it might be suggested that PKA may not affect the current separation protocol if it does not slow Nav1.8 repriming. However, if PKA does slow Nav1.8 repriming, a Nav1.8 selective blocker may be needed for the separation of SR and FR current (Jones et al., 2023). For PKC or other protein kinases, more experimental data on sodium channel repriming will be needed for further discussion. In contrast to PKA, several frequently used sodium channel inhibitors (carbamazepine, lacosamide, and lamotrigine) slightly slow the repriming of Nav1.7 and Nav1.6 (Hinckley et al., 2021). Moreover, vixotrigine induces a dramatic change in the repriming of both channels (Hinckley et al., 2021). Therefore, if a compound causes a slight change in repriming of both SR and FR currents, the current separation protocol may still work. Otherwise, the current protocol might not work for the compound.

The current study likely dissects out Nav1.7 from other TTX-S currents electrophysiologically in small DRG neurons. Nav1.7-like currents are differentially expressed among small DRG neurons between rats and mice and between IB_4_^+^ and IB_4_^−^ neurons. ProTx-II shows reduced efficiency and selectivity inhibiting endogenous Nav1.7-like currents. Paclitaxel selectively increased Nav1.7-like currents in IB_4_^−^ neurons. Nav1.7 contributes to a lower threshold of action potentials in simulated small DRG neurons. The electrophysiological dissection of Nav1.7-like current may provide a useful way to study the functional expression and pharmacology of Nav1.7 channels in DRG neurons. Moreover, combined with other approaches (pharmacology, neuronal staining, genetic reporter, patch-Seq, and so on), the electrophysiological dissection may improve feasibility and depth for studies on Nav1.7 and other TTX-S VGSCs in small DRG neurons.

## Data availability statement

The original contributions presented in the study are included in the article/supplementary material, further inquiries can be directed to the corresponding author/s.

## Ethics statement

The animal study was approved by Indiana University School of Medicine Institutional Animal Care and Use Committee. The study was conducted in accordance with the local legislation and institutional requirements.

## Author contributions

Z-YT: Conceptualization, Data curation, Formal analysis, Funding acquisition, Investigation, Methodology, Project administration, Resources, Supervision, Writing – original draft, Writing – review & editing. BW: Data curation, Formal analysis, Investigation, Methodology, Writing – review & editing. XS: Investigation, Methodology, Writing – review & editing. YZ: Conceptualization, Writing – review & editing. Y-HJ: Conceptualization, Writing – review & editing.
